# Modeling heterocyst pattern formation in cyanobacteria

**DOI:** 10.1186/1471-2105-10-S6-S16

**Published:** 2009-06-16

**Authors:** Ziomara P Gerdtzen, J Cristian Salgado, Axel Osses, Juan A Asenjo, Ivan Rapaport, Barbara A Andrews

**Affiliations:** 1 Centre for Biochemical Engineering and Biotechnology, Department of Chemical Engineering and Biotechnology, University of Chile, Av. Beauchef 850, Santiago 837-0448, Chile; 2 Department of Mathematical Engineering, Center for Mathematical Modeling (UMI 2807-CNRS), University of Chile, Casilla 170/3 Correo 3, Santiago, Chile

## Abstract

**Background:**

To allow the survival of the population in the absence of nitrogen, some cyanobacteria strains have developed the capability of differentiating into nitrogen fixing cells, forming a characteristic pattern. In this paper, the process by which cyanobacteria differentiates from vegetative cells into heterocysts in the absence of nitrogen and the elements of the gene network involved that allow the formation of such a pattern are investigated.

**Methods:**

A simple gene network model, which represents the complexity of the differentiation process, and the role of all variables involved in this cellular process is proposed. Specific characteristics and details of the system's behavior such as transcript profiles for *ntcA*, *hetR *and *patS *between consecutive heterocysts were studied.

**Results:**

The proposed model is able to capture one of the most distinctive features of this system: a characteristic distance of 10 cells between two heterocysts, with a small standard deviation according to experimental variability. The system's response to knock-out and over-expression of *patS *and *hetR *was simulated in order to validate the proposed model against experimental observations. In all cases, simulations show good agreement with reported experimental results.

**Conclusion:**

A simple evolution mathematical model based on the gene network involved in heterocyst differentiation was proposed. The behavior of the biological system naturally emerges from the network and the model is able to capture the spacing pattern observed in heterocyst differentiation, as well as the effect of external perturbations such as nitrogen deprivation, gene knock-out and over-expression without specific parameter fitting.

## Background

Cyanobacteria are blue-green algae, prokaryotic organisms capable of both obtaining energy by oxygenic photosynthesis and nitrogen fixation. They play a fundamental role in earth's carbon cycle as primary producers, and in the nitrogen cycle as nitrogen fixers [1]. These two processes are somewhat contradictory in nature, since the activity of the nitrogen fixing enzyme, nitrogenase, is dramatically reduced by the presence of oxygen, which is the end product of photosynthesis. Cyanobacteria evolved a mechanism for fixing dinitrogen (N_2_), an oxygen sensitive process, under aerobic conditions. Vegetative cells, which are the cells where oxygen is produced from photosynthesis, can differentiate into specialized non-dividing nitrogen-fixing cells called heterocysts. *Anabaena *and other filamentous cyanobacteria in the order of *Nostocales *grow in filamentous structures formed by photosynthetic vegetative cells. For some of these strains, under nitrogen-limiting conditions, vegetative cells differentiate into heterocysts at intervals along the filaments therefore generating a semi-regular spacing pattern [2]. This mechanism has made *Anabaena *a model organism for studies involving cellular differentiation and pattern formation in prokaryotes.

The primary known function of heterocysts is the fixation of dinitrogen, for which they require a reductant supplied by the vegetative cells since differentiated cells gain the ability to reduce nitrogen gas but lose the ability to fix carbon dioxide. Heterocysts have a deactivated photosystem II (a protective envelope that is semi permeable to oxygen) and higher respiration rates in order to consume the oxygen that was able to enter the cell. All these characteristics generate an anaerobic environment suitable for the operation of the nitrogenase enzyme and therefore, for nitrogen fixation [3,4]. Nitrogen fixed in heterocysts is transported along the filament, likely in the form of glutamine, and utilized by the whole population of cells [5]. In this scheme, heterocysts share nitrogen with vegetative cells receiving in turn carbon resources establishing a cooperative system [2].

Differentiation of vegetative cells into heterocysts is triggered by the absence of a fixed nitrogen source in the growth medium [6]. A large number of genes involved in the development and spacing of heterocysts have been identified, however the complete mechanism by which they interact is still unclear [7]. Interaction mechanisms, based on current knowledge and inferred interactions have been proposed [8-10].

The reduction in nitrogen levels rapidly enhances the activation of *ntcA*, a DNA-binding factor involved in the transcription of genes involved in nitrate and ammonium transport and assimilation, dinitrogen fixation and heterocyst development, including *hetR*, which is required for heterocyst differentiation [11,12]. Nitrogen levels are sensed by the cell's intracellular levels of 2-oxoglutarate. This intermediate from the Krebs Cycle accumulates in the cytoplasm upon nitrogen starvation, enhancing the DNA binding activity of NtcA [13]. There is evidence that NtcA binds to the promoter region of its own gene, suggesting that it regulates its own expression [14].

*hetR *plays a key role in the regulation of heterocyst differentiation. This gene appears to be indirectly activated by NtcA through intermediate signaling molecules, since none of its promoters contain the sequence to which NtcA binds [15]. The gene product HetR is a DNA binding protein that acts as a homodimer [16]. In the presence of nitrogen, *hetR *is transcribed at low levels in all cells. Following deprivation, *hetR *expression is induced in a process that requires *ntcA *expression and the presence of a functional *hetR *gene, indicating that *hetR *is also autoregulatory [17].

For the generation of the characteristic pattern observed upon heterocyst differentiation, besides the activation of genes required for heterocyst development, the genes responsible for regulating the position and spacing of heterocysts must be activated. Among the genes involved in the control of heterocyst spacing are *hetN*, *patA*, *patS *and a few others. The *patA *gene is transcribed at low levels in the presence of nitrogen, and transcription increases following nitrogen step-down in a similar pattern of expression to that of *hetR*. PatA is a protein whose structure suggests kinase activity, which is speculated to act indirectly on controlling heterocyst spacing since it does not contain a known DNA-binding motif. This protein is required for the enhancement of *hetR *transcription in developing heterocysts [9,15]. HetN regulates the activity of *hetR *likely through a secondary metabolite, therefore playing a regulatory role in the control of heterocyst formation [18].

PatS acts as a spacing regulator for heterocyst differentiation. This protein has been shown to block heterocyst differentiation when present in multiple copies or when over-expressed. PatS is produced early in heterocyst development and is believed to be processed (maybe by HetR) to release a pentapeptide capable of diffusing through the filament's periplasmic space to form an inhibition gradient around the developing heterocysts. This is supported by the finding that a synthetic peptide corresponding to the last five amino acids of PatS inhibits heterocyst development [19]. Studies have shown the feasibility of diffusion of this pentapeptide through nonspecific intercellular channels from cytoplasm to cytoplasm in *Anabaena *[20,21]. In addition, when *patS *is knocked-out filaments show an increased frequency of heterocysts and an abnormal spacing pattern [19]. It is speculated that PatS producing cells must be refractory to the influence of PatS, in order not to affect *hetR *gene expression, since it seems paradoxical how HetR can promote its own synthesis in the presence of the inhibitory pentapeptide derived from PatS. In fact it has been proposed that PatS is inactive in the cytoplasm of heterocysts and that an active peptide is released into the periplasm and transported to vegetative cells where it would act on HetR, or act on the transcription factors directly involved in the expression of *hetR *[15].

Several mathematical models have been developed in order to systematically explore and understand the system's behavior. Baker and Herman proposed a mathematical model for differentiation where heterocysts produce a substance that diffuses along the filament and inhibits the differentiation of vegetative cells into heterocysts. When its level in a cell drops below a threshold value, the cell differentiates into a heterocyst. The observed pattern of heterocysts is reproduced but the model shows high sensitivity to the parameters chosen, many of which are vague in terms of association to biological phenomena [22]. This model involves the production of an inhibitor by heterocysts and its diffusion along the filament where heterocysts develop if the inhibitors' concentration drops below a certain threshold. A subsequent work explains heterocyst differentiation by means of a competitive mechanism [23,24]. This mechanism involves an autocatalytic activator of differentiation produced by proheterocysts and an inhibitor produced in proportion to their degree of differentiation, which stops and even regresses proheterocyst differentiation and whose effect is reduced by adjacent vegetative cells. With this mechanism an irregular pattern will form on a filament where a maximum and minimum distance between heterocysts is observed [25]. Some of the elements in this proposed mechanism are in agreement with the underlying gene network currently identified to control differentiation of vegetative cells into heterocysts and addressed in this work. In this network the differentiation activator can be associated with the protein HetR, which enhances its own production, and the inhibitor with PatS, which inhibits the production of HetR [8].

Turing developed models that showed the possibility of obtaining patterns from an initially homogeneous distribution, from a reaction-diffusion system where two substances with different diffusion rates interact [26]. Gierer and Meinhardt showed that in morphogenesis a spontaneous pattern formation is possible if a locally short-range self-enhancing reaction associated with an activator which promotes its own production, is coupled with an antagonist that acts on a longer range [27,28]. Using this hypothesis Meinhardt showed that the *Anabaena *system satisfies the prediction of this model, considering a locally restricted activator HetR that self-activates nonlinearly (as a dimer) and a diffusible inhibitor PatS [29]. The pattern formed by this network has also been modeled and captured using L-system models where the network is described as a growing system with reaction-diffusion equations for antagonic activator and inhibitor molecules [30]. A population model was proposed by Pinzon and Ju to describe the effects of cellular activities and cultivation conditions on heterocyst differentiation at a culture-level [31]. More recently, Allard *et al*. proposed a dynamic model that considers random cell growth and division, as well as production, transport, and consumption of fixed nitrogen within the filament. In this model, nitrogen is the main trigger of cell differentiation and cell growth acts as a stochastic factor that can induce cell differentiation. Simulation results are in good agreement with experimental pattern distributions [32].

In this paper, a simple evolution mathematical model is presented, to study the pattern formation obtained upon heterocyst differentiation, based on the main regulatory elements and interactions of the gene circuit involved in differentiation: *ntcA*, *hetR *and *patS*. In contrast, previous models of the system are based on the protein network involved in heterocyst differentiation. The mathematical model proposed behaves as a discrete one-dimensional activator – inhibitor system, analogous to a continuous autocatalysis-inhibition model [26,27], but directly derived from the genetic network of the cyanobacteria. This model allows, not only to capture the stable states obtained by the system upon differentiation, but also the response of the system following modifications such as knock-out or over-expression of some of the elements and their effect on the patterns formed as well as the stable states achieved by the system. The underlying idea is the same as in previous models: there is an emerging periodic structure of localized heterocysts in the presence of a local autocatalysis (HetR) coupled with and a long-range inhibition (PatS). Nevertheless, the discrete model presented here has several important advantages when compared with the classical continuous autocatalysis-inhibition models. On one hand, the proposed model naturally arises from the interactions of the gene network involved in differentiation with a set of parameters that have direct biological interpretation. This is a bottom-up construction alternative to the typical top-down methodology of the continuous case where the parameters have to be adapted to each case from general set-up models.

Once the discrete model is established, it allows computing the patterns of the system upon differentiation and the frequency of the corresponding attractor basins. These are the equilibrium states of the system reached from a representative sample of random initial conditions. These types of calculations are a difficult task for the system of partial differential equations in continuous models. Moreover, since the model parameters have a biological meaning, the effect of modifications on the genetic network, such as knock-out or over-expression on the patterns achieved, can be tested. For this reason, the proposed model is suitable for experimental design and for exploring the pattern formation capabilities of systems that could have these pattern forming mechanisms. Finally, some variants of the model that include cell growth and death, or reduced binary on-off activity states can be implemented, but were not considered here for the sake of simplicity.

## Models and methods

### Modeling framework

A finite array of *n *genetically identical cells has been considered. A fixed number of *n *cells in the array, organized in a cyclical manner where each cell is connected to its right and left neighbors is used.

**Figure 1:**
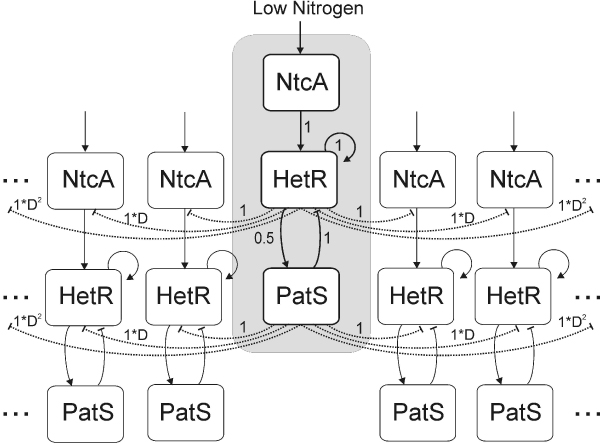
**Diagram of the network considered**. Cells are organized in a cyclical manner. Direct interactions are represented by solid lines and indirect interactions by dashed lines. Arrow heads indicate activation and vertical lines indicate inhibition. Numbers indicate the strength of the interactions considered among the elements of the network.

On each cell, the intracellular interactions are modeled by a gene circuit with three main interacting factors: *ntcA*, *hetR *and *patS*. The state of each cell in the array is characterized by the levels of each of these three components. This simple network is schematically represented in Figure 1, where each cell is represented by three nodes.

With this, an intracellular interaction matrix is obtained, given by

(1)

where the values of row *i *(*i *= *ntcA*, *hetR*, *patS*) represent the activation or inhibition strength that the proteins of the intracellular gene circuit exert on *i*. For instance, no protein acts on *ntcA *intracellularly (row 1) and *ntcA *acts on *hetR *with an activating strength of one. *C*_*Int *_summarizes all the network interactions that occur intracellularly for a single cell.

A reduction in nitrogen availability triggers the initiation of heterocyst differentiation mechanisms. Initially all cells behave in the same way, and the internal activity of each individual cell causes an inhibitory effect on two elements of the intracellular network of cells in its immediate neighborhood. Low external nitrogen levels trigger an increase in *ntcA *expression. NtcA leads to the transcription of *hetR*, which is self-activating. The increase in HetR levels enhances *patS *transcription to a lower extent than the other activation processes occurring intracellularly (represented with a strength of 0.5 in *C*_*Int*_), resulting in higher levels of the process pentapeptide PatS-5 (represented by PatS in Figure 1). It has been shown that this pentapeptide has an indirect inhibitory feedback effect on the expression of *hetR *in adjacent cells, by preventing the DNA binding of HetR and subsequent *hetR *up-regulation, therefore blocking heterocyst formation [16,19]. This is represented by a -1 in *C*_*Int*_. There is also an indirect long-term effect produced by the increased amount of nitrogen that becomes available once vegetative cells differentiate into heterocysts and start fixing nitrogen. This (indirect) effect is induced by HetR and sensed by the cell as low oxoglutarate levels, which in turn decrease the binding activity of NtcA [7]. Both PatS and the products of nitrogen fixation are the main signals determining the heterocyst pattern formed [6].

All these external interactions are represented in an extra cellular interaction matrix given by

(2)

This matrix shows the fact that *ntcA *expression is reduced by the indirect extracellular action of HetR and *hetR *expression is reduced by the action of extracellular PatS. *C*_*Ext *_summarizes all the interactions that occur in the network for a single cell due to its extracellular environment.

Transport of the inhibitory pentapeptide PatS-5 to adjacent cells through the periplasmic space is controlled by movement across the cell-cell interface and diffusion, and therefore it is dependent on the distance between cells. Similarly, the inhibition of *ntcA *by the indirect action of HetR is controlled by nitrogen diffusion. To account for these effects, a transport factor is introduced, *D *(*D *< 1) that affects the extracellular interaction matrix. All these network interactions (intracellular, extracellular, transport and diffusional effects) define the global interaction network *A *for the system. For instance, with *n *= 8

(3)

The matrix *A *has the intracellular interaction matrix (*C*_*Int*_) on its diagonal and the extracellular interaction matrix (*C*_*Ext*_) with a transport factor *D *on the secondary diagonals. The dependence of the effect of the extracellular interaction matrix with transport, represented by this transport factor, is reduced as the distance between cells increases. Therefore, its exponent increases in order as the distance from the neighboring cell increases and decreases as the distance (in number of cells) is reduced, as follows:

(4)

resulting a circulant (Toeplitz) type matrix *A*. The global interaction matrix *A *summarizes all the interactions that occur both intracellularly and extracellularly for an array of *n *cells with a gene network as the one shown in Figure 1.

The state of the system is associated with the protein levels of each of the elements in the network (*ntcA*, *hetR *and *patS*). These protein levels are normalized and associated to a real number between 0 and 1. The state of the system is therefore described by the levels of these factors on each cell of the array, represented by the vector *x*****∈ ℝ^3*n*^.

Starting from a random initial condition for the state vector that is biologically feasible for the system, that is, a random value between 0 and 1 for the normalized expression levels of each gene in the network, iteratively the system's equilibrium points were searched for following a perceptron type of rule associated to the nonlinear function *f*: ℝ → [0,1], defined as follows:

(5)

This function is used to determine if the expression of a particular element in the network is enhanced or reduced by the action of the other elements on the network. State transitions are considered to occur asynchronously, with one component of the state vector *x *being updated at a time in random order for the whole array of cells. This represents the fact that cells that differentiate are selected dynamically in response to nitrogen deprivation [4]. More precisely, the state vector *x *is updated as follows:

(6)

where *A*_*i *_is the *i*th row of the global interaction matrix *A*. In equation (6) the function *f*, which is used to determine enhancement or reduction of the activity of a particular element on the network *x*_*i *_in *t *+ 1, is dependent on the internal and external interactions for each cell in the previous state vector *A*_*i*•_*x*_*i*_(*t*), the external input associated with nitrogen levels *u*_*i *_(the *i*th element of the vector *u*), and the activation threshold *θ*. All cells in the system are considered identical since they all receive the same extracellular inputs and respond to the same thresholds. *θ *= 0.5 corresponds to the threshold level defined for the activation of each factor in the network. The vector *u*****∈ ℝ^3*n *^represents the effect on the intracellular network of the external input of nitrogen levels sensed by each cell, where

(7)

with *u*_0 _= 1 in the absence of nitrogen and *u*_0 _= 0 in its presence. Each triplet (1 0 0) of the vector *u*****is associated to the effect of an external input on an element of the network (*ntcA*, *hetR*, *patS*) for a cell in the array. A value of 1 indicates that nitrogen levels directly affect the first element on the network for each cell, *ntcA*.

**Figure 2:**
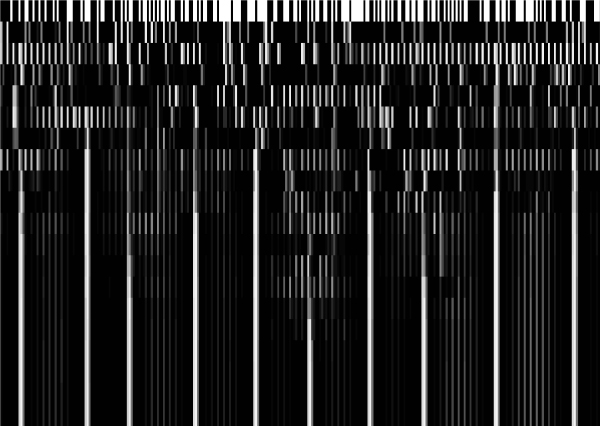
**Convergence plot of the network for an array of 100 cells starting from random uniformly distributed binary initial conditions, 0/black or 1/white for *ntcA*, *hetR *and *patS***. Each new row represents the expression levels after one random iteration starting from the state of the previous row. The system converges to one attractor where only some cells differentiate to heterocysts, represented by high values in HetR (the stable vertical lines formed in the figure). The average distance between heterocysts is approximately 10 with a transport factor *D *= 0.767.

The convergence state for heterocyst distribution achieved by the system from a random initial condition for the state vector is illustrated in Figure 2. As time (iteration) progresses the pattern formed is clearly defined.

## Results and discussion

### Effect of the transport of PatS

**Figure 3:**
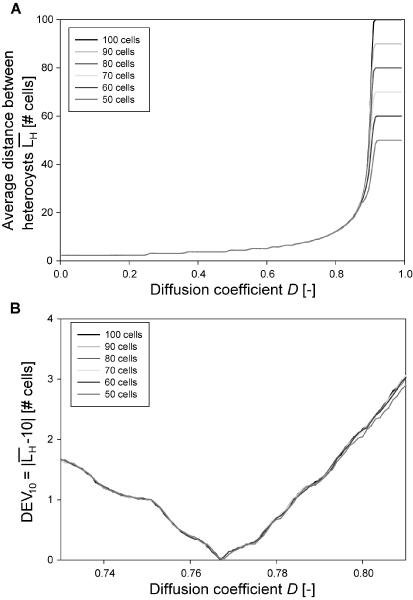
**Effect of the transport factor of PatS (*D*) on the average distance between heterocysts**. A: Simulations were performed using 1000 random uniformly distributed binary initial conditions, restricted to biologically feasible conditions.  is independent of the number of cells for *D *< 0.8. With higher values of *D*, the inhibitory activity of PatS to adjacent cells increases. For *D *close to 1 the average  corresponds to the total number of cells as only one cell differentiates, which corresponds to an average distance equal to the number of cells. B: Simulations were performed using 5000 random uniformly distributed binary initial conditions, restricted to biologically feasible conditions. The minimum value of this function is for *D *= 0.767 which gives a  of 10.

The analysis is started with the relationship between the transport factor and the system's behavior. Figure 3-A shows the effect of the transport factor *D *on the average distance between heterocysts . *D *was sampled in the interval [0, 1] in steps of 0.005 units and its effect studied for systems whose size ranges from 20 to 100 cells.  was calculated as the average distance between heterocysts obtained after the system converged for 1000 random initial conditions (it is assumed that using a larger number of initial conditions will not modify the results and discussion significantly). It was observed that noisier results are generated in the simulation of systems with a cell number lower than 50, due to the small size of the system and border effects. The relative importance of these anomalies decreases as the number of cells in the system increases. Based on this observation, the following analysis considers systems with more than 50 cells. Figure 3-A illustrates that the effect of *D *on the behavior of systems with different sizes is very similar, *i.e.*, it is independent of the number of cells. This indicates stability and shows good behavior for the model since  is not expected to vary with the number of cells when the transport factor is kept constant. The effect of *D *on  is very weak and almost linear until *D *= 0.700, indicating that low transport coefficient for PatS could cause heterocyst proliferation and, consequently, reduce . Starting from *D *= 0.700, the effect increased dramatically until *D *= 0.920 when heterocyst distance reaches a maximum. Higher values of *D *generate a scenario where the transport of PatS is greatly facilitated through the system. The saturation of the system with PatS inhibits the generation of heterocysts leading to a maximum in . It was found that these curves follow a power law before saturation. The coefficient of determination for L_H _following a power law with the equation  law, where *a*, *b *and *c *are the adjustable parameters, were of *R*^2 ^> 0.94 in all cases. According to Figure 3-A the behavior of the system, in terms of , could be controlled by means of the modification of *D*, the transport factor for PatS. Since the effects of changes in the transport of the PatS pentapeptide are difficult to test in an experimental setting, the usefulness of a mathematical model is clear. The transport factor *D *necessary to reach a specific  is shown in Figure 3-A. In particular, an average distance between heterocysts of ten cells ( = 10) is obtained when *D *≈ 0.7–0.8. More accurate values require a closer examination as shown in Figure 3-B.

The absolute value of deviation of  from 10, , for several values of *D *is shown in Figure 3-B. A lower value indicates a  closer to 10. In this case 5000 biologically feasible random initial conditions were used and *D *was sampled in the interval [0.730; 0.810] every 0.001 units. It was found that the transport factor (*D*_10_) that minimizes the deviation *DEV*_10 _in Figure 3-B is 0.767 for systems with 50 to 100 cells. Using this *D*_10 _gives systems with 's only 0.12% far from 10 (average error). The average of those transport factors is  = 0.767 ± 0.001. Based on this fact, 0.767 will be used as the transport factor for the following simulations.

### Average distance between heterocysts for the wild type

Table 1 shows the average distance between heterocysts, , and its standard deviation when a transport factor  = 0.767 is used for the wild type under nitrogen deprivation. It has been observed experimentally that although the average distance between heterocysts is *circa *10, it also displays some variations, which oscillate between 7 and 15. Standard deviations show that the mathematical model reproduces this characteristic distribution and suggests a narrow distribution of *L*_*H *_around .

**Table 1:** Average and standard deviation for the distance between heterocysts in the absence of nitrogen for the wild type , when *patS *expression has been knocked-out  and when *hetR *has been over-expressed . Simulations consider a transport factor *D *=  = 0.767 and 5000 biologically feasible randomly chosen initial conditions.

Number of cells			
100	10.010 ± 2.357	4.603 ± 3.368	6.161 ± 3.730
90	9.991 ± 2.356	4.611 ± 3.382	6.135 ± 3.725
80	9.977 ± 2.353	4.627 ± 3.382	6.119 ± 3.733
70	10.007 ± 2.355	4.627 ± 3.397	6.164 ± 3.727
60	9.999 ± 2.364	4.627 ± 3.398	6.115 ± 3.721
50	9.979 ± 2.364	4.612 ± 3.386	6.084 ± 3.691

**Figure 4:**
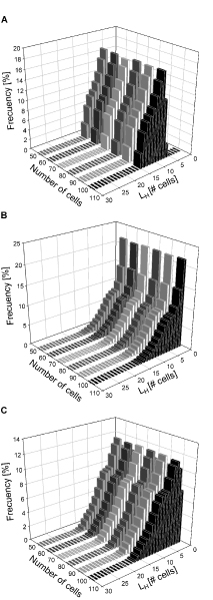
**Histograms for the distance between heterocysts in the absence of nitrogen**. A:  wild type.  does not follow a normal distribution. B:  when *patS *expression is knocked-out. C:  when *hetR *is over-expressed. All simulations were performed using 5000 random uniformly distributed binary initial conditions, restricted to biologically feasible conditions and considering *D *=  = 0.767, for systems with 50–100 cells. For easier comparison frequencies were normalized to the number of cells.

Histograms of *L*_*H *_for *D *=  are given in Figure 4-A. This figure shows that the distance between heterocysts, *L*_*H*_, for systems with different numbers of cells follows a very similar distribution. In general, the shape of this distribution is narrow, not symmetrical and does not follow a normal distribution (Kolmogorov-Smirnov test). The non-symmetrical shape of the histograms in Figure 4-A indicates that the frequency of observing an *L*_*H *_<10 is higher than the frequency of *L*_*H *_>10. In fact, for the case of the 100 cells system these frequencies are 47.8% and 39.2%, respectively. Even so, Figure 4-A shows that the distribution of *L*_*H *_is highly concentrated in the neighborhood of 10, a fact that has been observed experimentally. Our simulated results for the distribution of *L*_*H *_in wild type are consistent with the heterocyst spacing distribution observed experimentally by Yoon and Golden at 48 h with a number of vegetative cells between heterocysts ranging from 5 to 18 cells, peaking around 10 cells [6]. The model however does not capture the distribution observed at later culture times, where other factors such as cell death and decay may have an effect in the pattern distribution observed experimentally.

### Expression profiles between a pair of consecutive heterocysts

**Figure 5:**
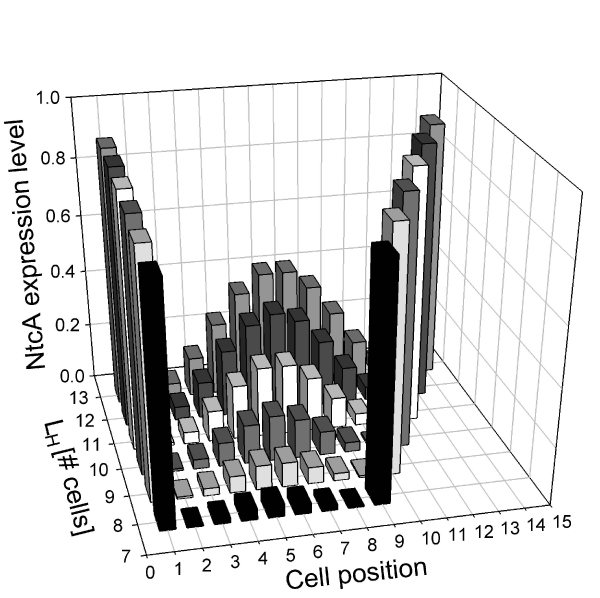
**Average *ntcA *expression profiles for cells located between two consecutive heterocysts with a distance **. Simulations were performed using 5000 random uniformly distributed binary initial conditions, restricted to biologically feasible conditions. This characteristic shape for *ntcA *activation profiles could not be achieved if the activation values were considered as boolean instead of taking continuous values in the [0,1] interval.

*NtcA *expression profiles for cells located between two consecutive heterocysts are shown in Figure 5. Given *L*_*H*_, *ntcA *expression levels for a cell located at the *i*th position in the array corresponds to the average expression for all cells located at the *i*th position between two consecutive heterocysts. Calculations were performed considering 5000 random initial conditions, *D *= , 100 cells, and *L*_*H *_= 8, 9,10,11,12 and 13. Note that, as shown in Figure 5, simulation results indicate that heterocysts are located at both ends of the array and have the highest *ntcA *expression levels. *ntcA *expression profiles in Figure 5 are bell-shaped curves centered equidistantly from both heterocysts. Therefore, maximum *ntcA *expressions are observed for the cell located at position L_H_/2, due to the symmetry of inhibitory effects from HetR and PatS (see Figure 1). An average variability of less than 11.1% was observed for the expression of *ntcA *at any given position.

The maximum *ntcA *expression levels change with *L*_*H *_and cell array size. In fact, as the distance between heterocysts increases, *ntcA *expression increases, as corroborated by experimental observations. Similarly, the inhibitory action of neighboring cells on *ntcA *expression is reduced as the distance between heterocysts increases. If the distance between heterocysts is high enough, oxoglutarate levels will be high to enhance *ntcA *expression on the cell located at position *L*_*H *_/2. Eventually, this expression will be high enough to enhance *hetR *expression and trigger cell differentiation into a heterocyst.

### Differentiation behavior as a result of external perturbations

The differentiating behavior of vegetative cells into heterocysts is triggered by external disturbances on nitrogen levels, and the cellular response to these disturbances based on its internal characteristics. In the presence of nitrogen, *ntcA *expression is reduced and differentiation does not occur. On the other hand, nitrogen absence will trigger *ntcA *expression and the chain of interactions leading to the differentiation of some cells (see Figure 1) [8].

The model presented in this paper is able to capture both of these behaviors, as has been shown in the previous sections. As an illustration, a case study is shown.

### Spacing distribution for the wild type in the presence and absence of nitrogen source

**Figure 6:**
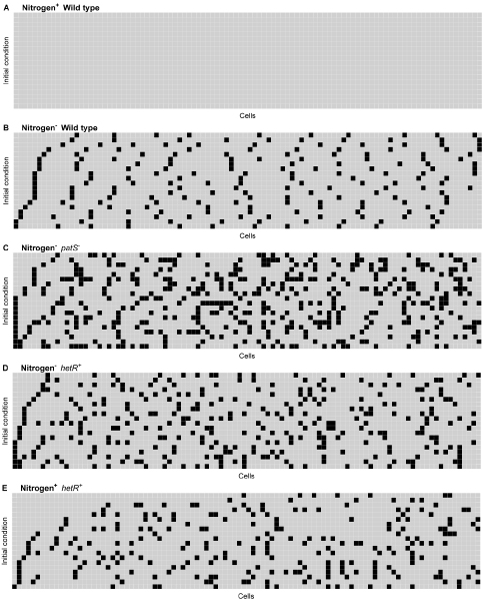
**Convergence states achieved by a 100 cell system from 20 biologically feasible randomly chosen initial conditions**. The position of each cell in the system is on the abscissa and on the ordinates the index of a random initial condition. A black rectangle indicates that, at the converged state, the cell in that position has been differentiated into a heterocyst: A: Wild type in presence of nitrogen. No heterocysts are formed. B: Wild type in the absence of nitrogen. On average 1/10 cells differentiates into a heterocyst. C: *patS *knock-out in the absence of nitrogen. Clusters of heterocysts are formed. D: *hetR *over-expressed in the absence of nitrogen. Clusters of heterocysts are formed at a lower frequency than for *patS *knock-out. E: *hetR *over-expressed in the presence of nitrogen. Differentiation into heterocysts at a lower frequency than for *hetR *over-expression in the absence of nitrogen.

A system of 100 cells was simulated, considering a transport factor  = 0.767, with 20 randomly chosen initial conditions. For this scenario, random uniformly distributed binary initial conditions were considered, restricted to biologically feasible scenarios. Since cell death is not considered in the model, *ntcA *and *hetR *must be considered as being turned off initially. Else the system would exhibit heterocysts initially and these heterocysts would remain throughout the simulation. Figure 6-A and 6B show the convergent state achieved by this system. In the presence of nitrogen (Figure 6-A), no cellular differentiation into heterocysts is observed. On the other hand, Figure 6-B shows that some cells differentiate into heterocysts in the absence of nitrogen, with an average distance of approximately 10 cells. In this case no restrictions are considered on initial conditions.

### Spacing distribution for *patS *knock-out and over-expression systems

It has been reported that genetic manipulation of the gene network involved in cell differentiation into heterocysts does change the differentiation pattern observed. Two extreme examples are the knock-out and over-expression of *patS *[19]. Simulation of the differentiation of an array of 100–50 cells with *D *=  with knocked-out expression of *patS *was performed. 5000 randomly chosen initial conditions were considered. Figure 6-C shows that for *patS *knock-outs the inhibitory effect of PatS is reduced, therefore decreasing the average distance between heterocysts and even allowing the formation of heterocyst clusters in some cases. Results in Figure 6-C are supported by *L*_*H *_histograms shown in Figure 4-B. These results indicate that knocking-out the expression of *patS *shifts *L*_*H *_distribution, significantly shortening the average distance between heterocysts, , compared to the wild type  (see Figure 4-A), as observed experimentally [19]. This is consistent with the results presented in a previous model, where mutation of PatS is reported to lead to a larger number of heterocysts [29]. Table 1 shows that  for *patS *knock-outs in systems with 100–50 cells is lower than 10 cells, and very similar for all systems. In fact, the frequency of observed  <10 is around 90.1%, almost twice as much as the wild type with normal *patS *expression. In addition, standard deviations for *patS *knock-out are higher than those for the wild type, indicating that knocking-out *patS *produces an increase in the variability of . Our simulated results for the distribution of *L*_*H *_in *patS *knock-outs are consistent with the heterocyst spacing distribution reported for a *patS *deletion strain 48 h after nitrogen step down. In this case the number of vegetative cells between hetercysts ranges from 2 to 14 cells, peaking around 5 cells. Heterocysts spacing distribution becomes broader compared to wild type, peaking towards small intervals. The model however, does not match the exact distribution observed experimentally after prolonged culturing, which becomes broader as the culture progresses [6] for the same reasons pointed out in the previous case.

Over-expression of *patS *was also simulated. To our knowledge, this condition has not been explored theoretically with a mathematical model for heterocyst differentiation. In this scenario, simulation results obtained with the present model exhibit a behavior equivalent to that shown in Figure 6-A, *i.e. *higher expression of *patS *completely inhibits differentiation into heterocysts.

### Spacing distribution for *hetR *knock-out and over-expression systems

HetR has been identified as a key factor in heterocyst differentiation. The effect of knocking-out and over-expressing *hetR *was also simulated, considering an array of 100–50 cells with *D *=  with 5000 randomly chosen initial conditions.

As expected, if *hetR *is knocked-out no heterocysts are formed [29]. However, *hetR *over-expression leads to heterocyst differentiation and the formation of heterocyst clusters as shown in Figure 6-D. The pattern obtained is similar to the one observed for *patS *knock-out. Figure 6-C and 6D respectively, show that the knock-out of *patS *has a stronger effect than the over-expression of *hetR *on differentiation into heterocysts, compared to the wild type in Figure 6-B. Results in Figure 6-D are supported by *L*_*H *_histograms shown in Figure 4-C. These results indicate that over-expression of *hetR *also shifts *L*_*H *_distribution, lowering the average distance between heterocysts , compared to the wild type (see Figure 4-A). This phenomenon has also been observed experimentally [18,33].

There are no previous reports on the theoretical exploration of the over-expression of *hetR *using a mathematical model for the gene network involved in heterocyst differentiation. Over-expression of *hetR *may also lead to the formation of heterocysts under normally repressing conditions when the gene is controlled by an external factor. In the present system, this corresponds to an over-expressed *hetR *scenario where biologically feasible initial conditions are only restricted to *ntcA *being off. Simulation results for this case are shown in Figure 6-E: heterocyst formation is observed even in the presence of nitrogen (23.91% less heterocysts than in the absence of nitrogen, Figure 6-D), which is consistent with the experimental observations reported [34].

### Final remarks

Additional factors such as cell growth and death were not considered as they do not affect directly the main activator-inhibitor mechanism. Three types of parameters are considered in this model; parameters related to transport (*D*), dynamics (matrix *A*) and a threshold (*θ*). The system shows to be robust for perturbations in the first two types of parameters, and not surprisingly, very sensitive to changes in *θ*, which results to be critical as it represents the limit that defines wether a particular element in the network is active or not. It is possible to capture the stable states of the system if the parameters are chosed adequately considering a biological interpretation of the network.  The model allows flexibility in the selection of values for the matrix A.

Although the parameters used have not been biologically validated, they retain biological meaning (e.g. >0 for activation, <0 for inhibition and relative magnitudes imply relative effects). In addition, given the characteristics of the implementation, the system can be extended to two and three dimensions.

It is important to note that this model, as other activation-inhibition type models, does not consider any assumptions regarding PatS. It is often said that it is essential that PatS remains inactive until processed in a neighboring vegetative cells for heterocyst-forming cyanobacteria to be able to succesfully activate *hetR *and therefore achieve their characteristic differentiation pattern. Here, no additional assumptions are considered on PatS, and in fact PatS may act on the cell on which it is produced and still a heterocyst develops, as long as the inhibitory effect is smaller than the counteracting activators effect acting on the same cell.

Additionally, no restrictions are imposed on the range of action of the differentiation activator HetR and the inhibitor PatS. Regardless of these assumptions, the model successfully captures the differentiation pattern observed experimentally for the initial stages of culture. This indicates that it may be possible for the system to achieve the characteristic pattern of heterocyst spacing, without a refractability condition on heterocysts to the action of PatS as discussed in most reviews. Even if PatS inhibited *hetR *self-activation in the cell where it is produced, pattern formation would be possible if the magnitude of the inhibitory effect on *hetR *was smaller than *hetR *self-activation.

One interesting thing is that the pattern formation characteristics naturally emerge from the system without data fitting, since the only parameter that was tuned is the transport factor *D*. This evolution model was not intended to capture the transient dynamics of the system, but to capture qualitatively the final spacing distribution observed. The simple gene network proposed is able to capture some of the main characteristics of the spacing pattern observed for heterocyst differentiation, with good agreement between simulation and experimental results both for wild type and for perturbations in the gene network.

## Conclusion

In this paper the differentiating behavior of cyanobacteria into heterocysts has been studied by means of a mathematical model. A simple gene network which is capable of capturing the complexity of the differentiation process in this cellular system is proposed.

Specific characteristics of the behavior observed experimentally in the presence/absence of nitrogen can be reproduced by the proposed system. The model presented explains how heterocyst-forming cyanobacteria counts to 10, the basic regulatory network elements required and the effect of diffusion on the spacing pattern observed. However, the reason why the system has evolved to this characteristic heterocyst distance remains to be explained. It appears that this has to do with the diffusional characteristics of the inhibitor PatS and the trade-off between resource requirements for heterocysts differentiation *vs*. their nitrogen fixing capabilities.

In particular, the model reports an average distance between two heterocysts equal to 10 cells, which is one of the most significant characteristics displayed by these systems. In addition, standard deviations of this magnitude were in accordance with the variability observed experimentally [2,8].

Specific details were also investigated, such as *ntcA *profiles between two consecutive heterocysts. In this case, simulations results also show a good agreement with experimental observations. Over-expression and knock-out of *patS *and *hetR *were also studied. The model is capable of simulating the qualitative behavior of heterocyst-forming cyanobacteria for these scenarios, capturing their distinctive characteristics, for instance: formation of high density clusters of heterocyst or complete inhibition of differentiation in the case of knocked-out and over-expression of *patS*, respectively. In the case of *hetR *the opposite behavior is observed: knocking-out *hetR *completely prevents the formation of heterocysts. On the other hand, over-expression of *hetR *leads to the formation of heterocyst clusters, but with a lower density and size than observed for *patS *knock-out. These results validate the proposed network structure.

## List of abbreviations used

Genes and gene products: *ntcA*: NtcA gene; *hetR*: HetR gene; *pat*: PatS gene; *pat*: PatA gene; NtcA: NtcA protein; HetR: HetR protein; PatS: PatS protein; PatA: PatA protein. Vector, matrices and others: C_*Ext*_: Extracellular interaction matrix; C_*Int*_: Intracellular interaction matrix; *A*: Global interaction matrix; *f*: Mapping function; *x*: Cellular state vector; *u*: External input vector; *θ*: Threshold levels vector; *D*: Normalized transport factor.

## Competing interests

The authors declare that they have no competing interests.

## Authors' contributions

ZPG proposed the genetic network to be considered as a system, designed *in silico *experiments, performed biological interpretation of the results, drafted and revised the manuscript. JCS programmed the algorithm, performed results analysis, designed *in silico *experiments and drafted the manuscript. AO proposed the discrete autocatalysis-inhibition model and programmed the main core of the algorithm used. All authors participated in development of the methods and preparation of the manuscript. All authors read and approved the final manuscript.
